# Developing Integrated Old Age Psychiatry and Care of the Elderly Medicine Services for People With Parkinson’s Disease: Service Development and Evaluation

**DOI:** 10.1192/bjo.2025.10517

**Published:** 2025-06-20

**Authors:** Jennifer Parker, Seona Duroux, Thushanthy Prasath, Catherine Penman, Emma Stratton

**Affiliations:** 1 Avon and Wiltshire Mental Health Partnership NHS Trust, Bristol, United Kingdom; 2 University Hospitals Bristol and Weston NHS Foundation Trust, Bristol, United Kingdom

## Abstract

**Aims:** Parkinson’s disease is a neurodegenerative condition with a lifetime risk of 2.7%, with a rise in prevalence expected in line with an ageing population. Whilst characteristically associated with motor symptoms, it is a multi-system disease with neuropsychiatric sequelae which are frequently missed by non-psychiatric specialists. Patients face barriers to access psychiatric services.

We describe a 15-month pilot of a novel integrated service for people with Parkinson’s disease in the Bristol Royal Infirmary. A monthly joint outpatient clinic was established whereby old age psychiatrists from the later life liaison psychiatry team and geriatricians saw patients within the same appointment. Additionally, we collaborated for weekly multidisciplinary team (MDT) meetings, inpatient reviews and wider liaison. Our aim was to develop a holistic integrated service with the hypothesis that this would offer value to our joint patient cohort and the wider healthcare service.

**Methods:** Patients were identified through triage of outpatient referrals, as inpatients and at MDT meetings. Clinical outcomes from the integrated clinic were measured using the Clinical Global Impressions (CGI) scale. Patient and professional quantitative feedback was gathered. Hospital admission data was measured against baseline admission rates for similar outpatient groups.

**Results:** Between November 2023 and January 2025, eleven integrated clinics were run and 33 patients attended; some patients were seen on multiple dates. The rationale for integrated working included new psychiatric symptoms (17%), pre-existing psychiatric diagnosis complicated by dopamine treatment (28%), cognitive conditions (39%) and complex psychotropic prescribing (33%). Major treatment outcomes included medication adjustment (78%), diagnostic reformulation and psychological therapy provision. There was a clear positive trend in CGI data showing benefit to patients, with overwhelmingly positive patient and professional feedback. Formal analysis of data looking at hospital admissions was inconclusive – but case series analysis shows examples of admission avoidance.

**Conclusion:** We believe the development of this service shows that close working between psychiatrists and physicians enhances patient care in Parkinson’s disease. Our integrated service is acceptable and beneficial for patients. It is valued by professionals and appears to be cost-effective through medication rationalisation and admission avoidance. In terms of future direction, we have applied for additional funded psychiatrist hours from the Trust to ensure sustainability of the clinic and are in the process of developing linked psychological therapy and clozapine prescribing services as a result of the success of the pilot clinic.

